# Five critical blind spots limiting translational progress in primary sclerosing cholangitis

**DOI:** 10.1097/HC9.0000000000001072

**Published:** 2026-10-19

**Authors:** Martin Cornillet

**Affiliations:** Department of Medicine, Center for Infectious Medicine (CIM), ANA Futura, Karolinska Institutet Campus Flemingsberg, Huddinge, Sweden

Primary sclerosing cholangitis (PSC) is a progressive inflammatory and fibrotic disease of the bile ducts that can lead to cirrhosis, liver failure, and cholangiocarcinoma (CCA).^1^,^2^,^3^ Despite major advances in single-cell and spatial biology, multiomics, imaging, and international cohort development, PSC has not experienced a therapeutic revolution. I propose that this translational gap reflects critical deficiencies in human translational infrastructure: many of the most informative patients, tissues, and disease stages remain understudied. In this Editorial, I discuss 5 blind spots that continue to limit progress and propose strategies to overcome them through human-centered translational research.

## BLIND SPOT 1: THE MISSING EARLY DISEASE WINDOW

Most mechanistic investigations involve established disease, advanced fibrosis, transplantation, or CCA. Consequently, concepts of PSC pathogenesis are largely reconstructed from late consequences, although the disease probably develops silently for years before diagnosis.

Historical liver and colon biopsy archives provide a largely overlooked record of earlier PSC. Advances in spatial transcriptomics, multiplex imaging, digital pathology, and proteomics now enable deep characterization of formalin-fixed tissues collected years before transplantation, cancer, or other major events.

Inflammatory Bowel Disease cohorts offer another opportunity. Magnetic Resonance Cholangiopancreatography screening of Inflammatory Bowel Disease cohorts has already identified previously unrecognized PSC in a small number of studies, demonstrating the feasibility of this approach. However, these studies were not designed to investigate the biology, mechanisms, pathogenesis, or biomarkers of disease initiation. Prospective Inflammatory Bowel Disease cohorts combining longitudinal blood, stool, intestinal biopsies, and imaging could therefore become platforms for studying the earliest biological stages of PSC.

Routine ERCP also permits repeated sampling through brushings, bile aspirates, and cholangioscopy-guided biopsies, potentially revealing longitudinal epithelial, immune, fibrotic, and clonal changes. Recurrent PSC provides a complementary model: recurrence develops in a previously unaffected donor liver from a known starting point, with serial blood, biopsies, and paired native and graft tissues available. Together, these resources could shift PSC research from reconstructing initiation retrospectively to observing it more directly.

## BLIND SPOT 2: UNDERUTILIZATION OF ROUTINELY COLLECTED CLINICAL MATERIAL

PSC generates substantial biologically informative material through routine care. The principal infrastructure gap is not simply access to samples, but coordinated collection, annotation, biobanking, and longitudinal linkage. Tissue archives, serial blood, biliary specimens, transplant material, and imaging datasets remain fragmented across centers.

There are important reasons why these resources have not been fully exploited. Historical material may lack research consent or standardized processing; preanalytical variation and incomplete metadata can complicate analyses; clinically indicated sampling creates spectrum and indication bias; and clinical practice changes over time. Transplantation is particularly complex because recurrent PSC can be difficult to separate from ischemic cholangiopathy, infection, rejection, and other graft injuries. These limitations require careful phenotyping, harmonized metadata, quality control, and validation rather than abandonment of the material.

Imaging illustrates the same opportunity. ANALI and DiStrict scores and quantitative Magnetic Resonance Cholangiopancreatography approaches have moved PSC imaging beyond purely descriptive assessment.^4^ The remaining translational gap is that imaging phenotypes are rarely integrated with the underlying epithelial, immune, stromal, fibrotic, or molecular biology, and are not yet routinely validated for clinical decisions. Linking routinely generated samples and imaging to biological measurements could transform fragmented clinical material into a longitudinal translational infrastructure.

## BLIND SPOT 3: PSC IS MORE THAN A LIVER DISEASE

That PSC involves more than the liver is not a new concept. The gut-liver axis, HLA-associated susceptibility, immune dysregulation, and extrahepatic manifestations have substantial literature. The blind spot is the mismatch between this accepted concept and the biology actually studied. The vast majority of tissue-based mechanistic studies still focus on the liver, often in advanced or end-stage disease; fewer examine the intestine, and remarkably few directly interrogate other potentially relevant immune, neural, endocrine, or lymphoid compartments.

PSC may therefore be better investigated as a disorder of interorgan homeostasis involving the intestine, biliary tree, immune system, microbiome, and neuroendocrine networks.^5^,^6^,^7^,^8^ This perspective may also help reconcile a central paradox: the coexistence of autoimmune disease, suggesting impaired self-tolerance, and a high risk of CCA, suggesting defective tumor immunosurveillance. Yet central immune and lymphoid compartments remain largely unexplored. Recurrent PSC is particularly informative because the disease develops in an initially healthy donor liver exposed to persistent recipient-derived immune, intestinal, microbial, and alloimmune influences. The biliary tree may not be the origin of disease, but the organ in which systemic disturbances ultimately converge. The challenge is therefore not only to study the liver more deeply, but to reconstruct this network across organs, time, and scales of human biology.

## BLIND SPOT 4: THE INTERNATIONAL BIOMARKER COORDINATION GAP

PSC biomarker research has generated circulating, imaging, microbiome, and multiomic candidates, yet few have progressed to clinical implementation. The problem is not biomarkers in general, but qualification for a defined context of use. A CCA surveillance test, a prognostic or enrichment biomarker for trial selection, a pharmacodynamic marker, and a surrogate endpoint require different evidence.

The FDA-NIH BEST framework provides terminology for defining biomarker roles, while the FDA Biomarker Qualification Program and EMA Qualification of Novel Methodologies provide pathways for regulatory qualification. PSC studies should therefore specify the context of use early and build validation accordingly. The emergence of large prospective resources, including the SUPRIM cohort,^9^ International PSC Study Group cohorts, UK-PSC, the Scandinavian PSC Biobank, the WIND project, and other national and regional initiatives, now provides the scale required for independent validation across populations, disease stages, sample types, and clinical settings. Coordinated qualification programs, rather than repeated isolated discovery studies, are needed to determine which biomarkers are sufficiently robust and actionable for surveillance, trial enrichment, response assessment, or endpoints.

## BLIND SPOT 5: THE HUMAN BILE DUCT REMAINS A MISSING ORGAN

The primary target tissue of PSC remains incompletely characterized. The gap is particularly acute for the large intrahepatic and extrahepatic ducts, peribiliary glands, and biliary-associated immune, stromal, vascular, and neural compartments across anatomical levels and age. Liver atlases have largely focused on parenchyma, leaving limited healthy reference data for the structures most directly affected by PSC.

Without such reference maps, disease-associated states are difficult to distinguish from normal anatomical and biological variation. Single-cell and spatial transcriptomics, multiplex imaging, deep proteomics, and organoid models, combined with healthy donor tissue, explants, transplantation material, and ERCP-derived samples, could establish reference maps spanning health, inflammation, fibrosis, and carcinogenesis. Perhaps the greatest paradox in PSC is that the field continues to search for disease mechanisms in an organ whose normal biology remains largely unexplored.

## FUTURE DIRECTIONS AND CONCLUSIONS

These 5 blind spots share a common theme: PSC research may not lack technologies, hypotheses, or samples, but fails to integrate its most informative human biology. Progress will depend on studying the right patients, tissues, and disease stages; making routine clinical material analytically usable; connecting biology across organs; qualifying biomarkers for explicit clinical purposes; and establishing reference biology for the human biliary tree. This shift could transform fragmented observations into actionable insight, enabling earlier disease interception, reliable biomarkers, and mechanism-guided care across the PSC patient journey.
